# Comparison of JB4 plastic resin and standard paraffin methods on student performance and student perspectives in digital histology education: A randomized controlled study

**DOI:** 10.1002/ase.70213

**Published:** 2026-03-08

**Authors:** Zeynep Deniz Şahin İnan, Ezgi Ağadayı

**Affiliations:** ^1^ Department of Histology & Embriology, Faculty of Medicine Sivas Cumhuriyet University Sivas Türkiye; ^2^ Department of Medical Education, Faculty of Medicine Sivas Cumhuriyet University Sivas Türkiye

**Keywords:** histology/education, medical students, microscopy/methods, paraffin embedding, synthetic resins

## Abstract

This randomized controlled study compared the effectiveness of histological preparations embedded in glycol methacrylate‐based JB4 plastic resin with traditional paraffin blocks in digital histology education. A total of 297 second‐year medical students at Sivas Cumhuriyet University participated. After a theoretical lecture on epithelial histology, students completed a pre‐test and an immediate post‐test before being randomly assigned to JB4 or paraffin groups. Both groups received laboratory instruction with their respective preparations displayed on a large screen, followed by a post‐intervention test, a drawing‐based performance assessment, and a follow‐up test administered 3 months later. Quantitative results showed that the JB4 group achieved significantly higher scores in the post‐intervention test compared to the paraffin group (6.0 ± 2.1 vs. 4.7 ± 1.7, *p* < 0.001) and exhibited lower knowledge loss in the follow‐up test (−1.2 ± 1.3 vs. −2.5 ± 1.7, *p* < 0.001). No significant difference was observed between groups in drawing‐based performance (*p* = 0.183), although female students outperformed male students in practical drawing (*p* = 0.001). Qualitative findings from focus group interviews revealed that students valued JB4 sections for their clarity, visibility of details, and preservation of cellular structure, while preparation difficulty, excessive detail, and higher cost were noted as disadvantages. Overall, the results suggest that JB4 sections may improve both immediate and long‐term learning outcomes compared to paraffin sections. Despite challenges related to preparation workload and cost, the use of JB4‐derived digital materials represents a promising complementary approach to enhancing the quality and accessibility of histology education.

## INTRODUCTION

Medical histology is a critical and essential component of medical curricula. The study of normal tissue morphology and cellular details is also known as microscopic anatomy. This training is divided into general and specialized histology and is based on traditional strategies of intensive theoretical lectures and practical training. The aim of this training is to give students experience in identifying tissues after acquiring theoretical knowledge.^1^ Practical competence in histology is traditionally acquired through the use of optical microscopy, a process that involves the preparation of tissue samples embedded in paraffin blocks and subsequent staining. Traditional microscopes are indispensable tools in histology laboratories.^1^, ^2^ In this context, Pratt (2009) emphasized that despite increasing technological integration in medical education, histology remains a foundational discipline for clinical reasoning and diagnostic competence, warning against the risk of marginalizing microscopic anatomy in medical curricula. Despite the advancements in real‐time imaging technology, histological sectioning and staining remain essential techniques for visualizing cellular morphology in tissues. Histo‐techniques are also important for understanding the progression of tissue development or addressing phenotypes related to pathological conditions.^3^, ^4^

In recent years, the integration of digital tools has become increasingly central to medical education in disciplines where visual analysis plays a critical role, such as histology and pathology. Virtual microscopy applications have reduced spatial constraints and made learning processes more interactive by providing access to histological preparations through multiple devices.^5^ In studies comparing virtual and traditional applications in histology teaching, it has been shown in many studies that they increase student performance and support learning.^1^, ^6^, ^7^

Advances in histotechnical methods have enabled the observation of cellular details with greater clarity and preservation. The JB‐4 (glycol methacrylate‐based resin) technique provides an alternative to the traditional paraffin method, offering a high level of preservation of cell morphology. JB4 sections are particularly notable for their tissue integrity and staining clarity.^3^, ^8^

In the field of histology education, only a limited number of studies have examined the impact of the paraffin method on learning outcomes, depending on the preparation type in both classical microscope and virtual microscopy environments. This study offers a distinctive contribution to the existing literature by comparing the effects of JB4 and paraffin sections on student learning levels, knowledge retention, and student perspectives in a virtual microscopy environment. A review of the literature revealed no studies that had evaluated the effect of preparations prepared with the JB4 technique on the learning process.

The objective of this study was to compare the histologic preparations prepared by embedding in glycol methacrylate‐based JB‐4 plastic resin, which provides excellent preservation of tissue and cell morphology, with those prepared by embedding in routine paraffin blocks. These comparisons were made to enhance the education of second‐year medical students.

## METHOD

### Participants

Participants were recruited from the second grade of Sivas Cumhuriyet University Faculty of Medicine during the 2024–2025 academic year. Exclusion criteria were determined as follows: previous histology education, unwillingness to participate, and failure to complete the necessary training and tests at the stages of the study. Histology education is incorporated into the curriculum in the second year of this faculty.

### Design

The effect of the type of education on knowledge was designed as a randomized controlled study. Student perspectives were assessed using open‐ended questions and focus group interviews.

All students were given the Pre‐Test at the same time in the same room. Without dividing the students into groups, researcher Şahin İnan gave a 2‐h theoretical lecture on epithelial histology. After the lecture, all students were given the Immediate Post‐Test. One week after the theoretical lecture, all students examined paraffin preparations under a microscope in the laboratory for 1 h, with each student having access to a microscope. Subsequently, students were allocated into two groups, ‘Paraffin’ and ‘JB4,’ through stratified random sampling based on their pre‐test scores. This procedure was conducted by an independent statistician, who generated a random number between 0 and 1 for each student using the ‘=RAND()’ function in Microsoft Excel 365. Students learned their groups before the implementation. The paraffin group was taught epithelial histology by researcher Şahin İnan, who projected paraffin preparations onto a large screen. The JB4 group was taught the same topics by the same researcher, who projected JB4‐stained preparations onto a large screen. After this application, all students were given a Post‐Intervention Test. Three months later, a Follow‐Up Test was administered to assess the retention of information. The same 10 questions were repeated in all tests (Pre‐Test, Immediate Post‐Test, Post‐Intervention Test, and Follow‐Up Test). These questions were prepared to include the endothelium, connective tissue fibroblasts, connective tissue epithelioids, renal Bowman's capsule epithelium, heart muscle, pancreatic cells, follicular structure, uterine gland structure, mitotic division stages, and mast cells. They were designed in a multiple‐choice format with five options so that students could correctly identify these structures from paraffin images. The difficulty indices of the questions were calculated for each exam. After the laboratory classes, students were given white paper and asked to draw and label the endothelium, connective tissue fibroblast, connective tissue epithelioid, renal Bowman's capsule epithelium, cardiac muscle, pancreatic cells, follicular structure, uterine gland structure, mitotic division stages, and mast cells. These drawings were also scored using a checklist. The test questions and checklist were prepared by the researchers.^9^ The images were selected from the Department of Histology & Embryology archive.^10^, ^11^

In the study, a sample was selected from students in the JB4 group who volunteered for focus group interviews to answer open‐ended questions using convenience sampling. The number of focus groups was determined based on data saturation as determined by the researchers. In addition to these questions, the researchers were able to obtain in‐depth information by asking different questions as needed based on the participants' responses. To ensure validity and reliability, two researchers participated in the interviews. While one researcher conducted the interviews, the other took notes. The researchers' notes and recordings were evaluated by both researchers. The transcripts of the researcher who conducted the interview and the documents of the researcher who took notes were compared. Open‐ended interview questions:

- What do you think are the advantages of JB4‐stained preparations?
- What are the disadvantages of JB4‐stained preparations?
- For histology practical training, would you prefer JB4‐stained sections or paraffin sections? Why?

**FIGURE 1:**
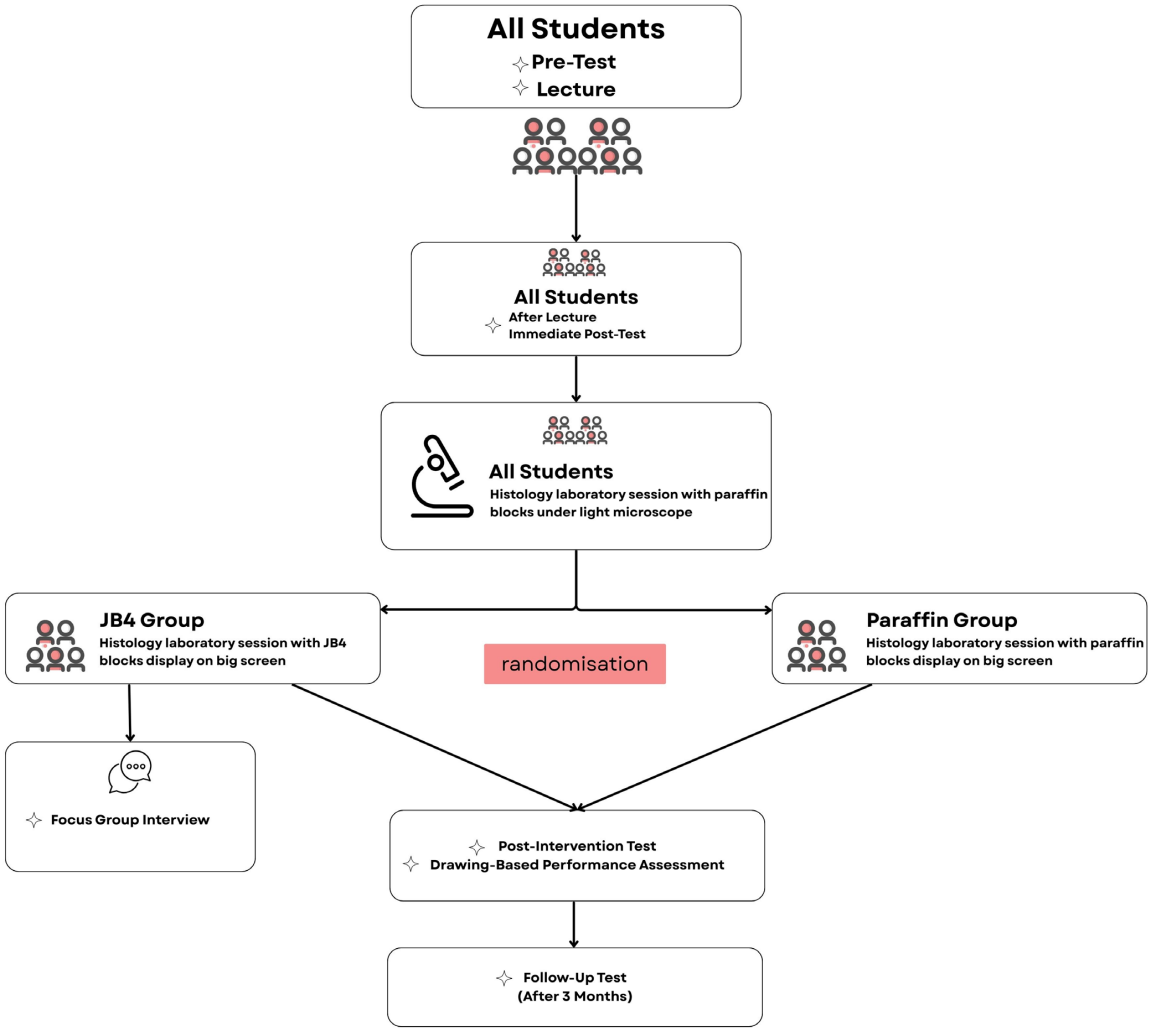
The research design.

The research design is schematized in Figure 1.

The outcome sets in the study are knowledge and student perspectives. Multiple‐choice tests and checklists were used to evaluate the outcome measurement of “knowledge” in the study. The outcome measurement of “student perspectives” was investigated in depth through qualitative research. The permanence of “knowledge” was also evaluated in the study.

### Light microscopy methods

All preparations were obtained from the Histology‐Embryology Laboratory of Sivas Cumhuriyet University. The specimens in the laboratory archive were prepared using paraffin and JB4 techniques.

Paraffin method: The specimens were fixed in 10% buffered neutral formaldehyde for 24–48 h at room temperature. The fixed tissues were dehydrated using a series of ethanol solutions with concentrations of 50%, 70%, 80%, 96%, and 100%. This process was followed by the use of xylene and hot paraffin to ensure complete tissue tracking. The tissues were embedded in paraffin blocks, and 5–7 μm thick sections were taken with a microtome. Following physical and chemical deparaffinization, the sections were rehydrated using a series of decreasing alcohol solutions. They were then stained with hematoxylin and eosin (H&E). Excess hematoxylin was removed with acid alcohol, and the sections were mordanted with ammonia. Following the eosin staining, the dehydration process was repeated. The preparations were then stored in xylol overnight to achieve transparency and subsequently sealed with Entellan. Both paraffin blocks and HE‐stained coverslips were stored in the departmental archive.^12^, ^13^

JB4 method: Tissues were fixed in 3% glutaraldehyde fixative for a minimum of 48 h at room temperature, followed by washing in phosphate buffer for 3 h. Following the washing process, the tissues were meticulously cut into small pieces. Then, they were dehydrated using a series of ethanol solutions with concentrations of 70%, 80%, 90%, 96%, and 100%. This process was carried out in a specialized tissue tracker. Subsequently, the tissues were stored in a catalyzed JB4 Solution A for an overnight period. The following day, the tissues were embedded in glycol‐methacrylate‐based JB4 resin and stored at +4°C for 3 days to allow for polymerization. For light microscopic examination, 1–1.5 μm thick sections were taken from these blocks, and Acid Fuchsin–Toluidine Blue stain was applied to show the general morphology. The preparations and blocks were then archived.^8^

**FIGURE 2:**
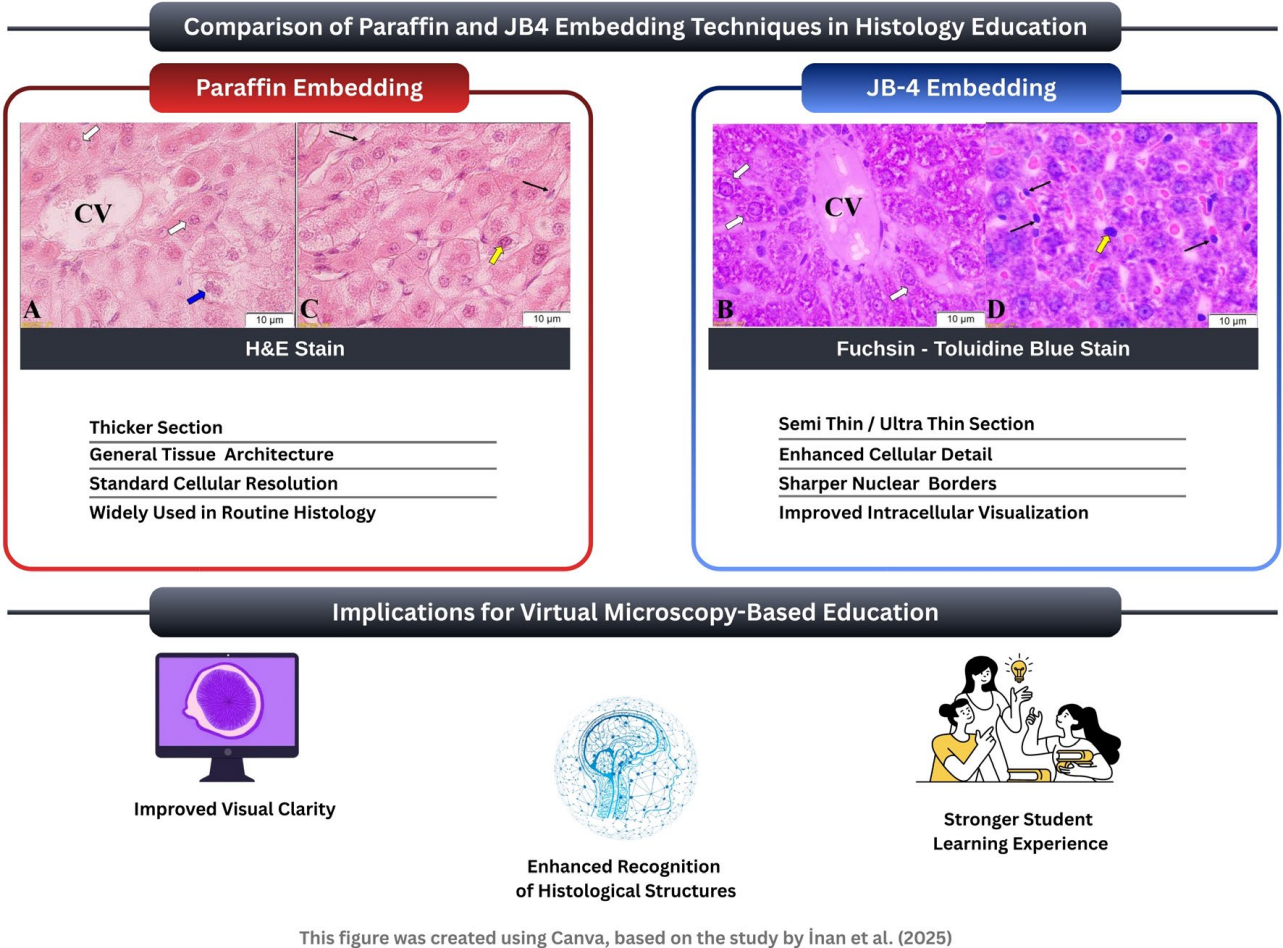
Representative histological images illustrating the visual and morphological differences between paraffin‐embedded and JB‐4–embedded liver tissue sections used in a virtual microscopy–based histology education setting. Panels (A) and (C) show liver sections embedded in paraffin and stained with H&E, demonstrating overall tissue architecture with relatively thicker sections and standard cellular resolution. Panels (B) and (D) show liver sections embedded in JB‐4 (glycol methacrylate–based resin) and stained with Fuchsin–Toluidine Blue, exhibiting semi‐thin sections with enhanced cellular detail, sharper nuclear boundaries, and improved visualization of intracellular structures. All panels (A–D) were captured at 100× magnification, with panels (C) and (D) providing higher‐detail views of the hepatic parenchyma. In these images, the central vein is marked by white arrows, while binucleated hepatocytes are indicated by blue arrows; Kupffer cells appear as larger, more irregularly shaped elements highlighted by yellow arrows, and sinusoidal endothelial cells lining the sinusoidal walls are denoted by thin black arrows. The schematic summary highlights key technical differences between paraffin and JB‐4 embedding methods and their potential educational implications, including improved visual clarity, enhanced recognition of histological structures, and a stronger student learning experience in virtual microscopy environments. This figure was adapted from the comparative findings reported by Sahin Inan et al.^14^

Figure 2 highlights key differences in section thickness, cellular and nuclear detail, and staining contrast between the two preparation methods, thereby illustrating how these technical differences may influence histological interpretation and learning in a virtual microscopy‐based educational setting.

### Statistical analysis

The data obtained in the study were analyzed using SPSS 22.0 (IBM Corp., Armonk, NY, USA) software package. Descriptive statistics were presented as mean, standard deviation, median, minimum, and maximum values. The Mann–Whitney *U* test was used for comparisons between two independent groups. To demonstrate the effect size of nonparametric comparisons, Rank‐Biserial Correlation (*r*_s_) was calculated. In the study, the effects of gender and group variables on exam results were analyzed using two‐way ANOVA. Spearman correlation analysis was conducted to determine the relationships between variables. For the evaluation of changes over time, the Friedman test was applied, and post‐hoc analyses were performed in cases where significant differences were identified. Post‐hoc differences were assessed using the Wilcoxon signed‐rank test. A significance level of *p* < 0.05 at a 95% confidence interval was considered statistically significant in all analyses.

### Ethical approval

The study was approved by Sivas Cumhuriyet University Health Sciences Research Ethics Committee (Protocol No: 2024/09‐42; Date: 19.09.2024). Written informed consent was obtained from all participants.

## RESULTS

In our study, out of a population of 326 second‐year students, 297 participated in the Pre‐Test and Immediate Post‐Test, yielding a response rate of 91.1%. Students' prior knowledge before the lecture was assessed using the Pre‐Test. The mean Pre‐Test score was 2.1 ± 1.8 (min: 0–max: 7). Following the theoretical lecture, the Immediate Post‐Test was administered. A significant but weak positive correlation was found between the Pre‐Test and Immediate Post‐Test scores (*r* = 0.29, *p* < 0.001). Based on their Immediate Post‐Test scores, students were randomly assigned to the JB4 and paraffin groups. The Immediate Post‐Test score of the JB4 group was 3.8 ± 1.3, while that of the paraffin group was 3.6 ± 1.7 (*p* = 0.147).

All students then attended a laboratory session, during which paraffin blocks were distributed for examination under a light microscope. Subsequently, epithelial tissue histology from paraffin sections was demonstrated on a large screen for the paraffin group, whereas epithelial histology from JB4‐stained sections was displayed for the JB4 group. At the end of the session, a Post‐Intervention Test was administered. A total of 264 students participated in this test, corresponding to a response rate of 81.0%. A statistically significant difference was found between the two groups in the Post‐Intervention Test (*p* < 0.001). The mean score in the JB4 group was 6.0 ± 2.1 (min: 1–max: 9), while in the paraffin group it was 4.7 ± 1.7 (min: 1–max: 8). After the session, students were asked to draw histological preparations, which were evaluated using a checklist. A total of 280 students participated in this test, yielding a response rate of 85.9%. The mean Drawing‐Based Performance Assessment score was 5.6 ± 1.8 (min: 0.75–max: 9) in the JB4 group and 5.1 ± 2.3 (min: 0.25–max: 9) in the paraffin group, with the difference between the groups not reaching statistical significance (*p* = 0.183).

Table 1 presents the response rates of the students across the groups.

**TABLE 1:** Group‐wise distribution of student response rates.

Type of assessment	Number of participants	Response rate (%)
Pre‐test		
Paraffin	167	100
JB4	130	100
All students	297	91.1
Immediate post‐test		
Paraffin	167	100
JB4	130	100
All students	297	91.1
Post‐intervention test		
Paraffin	134	80.2
JB4	130	100
All students	264	81.0
Follow‐up test		
Paraffin	46	27.5
JB4	36	27.6
All students	82	25.2
Drawing‐based performance assessment		
Paraffin	154	92.2
JB4	126	96.9
All students	280	85.9

*Note*: Pre‐test: Baseline knowledge assessment before the intervention (initial status). Immediate post‐test: Knowledge assessment immediately after the lecture (short‐term gain). Post‐intervention test: Assessment after group‐specific visual material (paraffin/JB4 slides). Follow‐up test: Retention assessment 3 months later (long‐term retention). Drawing‐based performance assessment: Practical skill assessment through drawing or schematic representation.

To assess knowledge retention, a Follow‐Up Test was administered 3 months later, with 82 students participating (response rate: 25.2%). The mean difference between the Post‐Intervention Test and the Follow‐Up Test scores was −1.2 ± 1.3 in the JB4 group and −2.5 ± 1.7 in the paraffin group, indicating a statistically significant difference (*p* < 0.001). Longitudinal changes in students' performance were analyzed using the Friedman test, and the results are presented in Table 2.

**TABLE 2:** Time‐dependent progression analysis of students.

	Pre‐test	Immediate post‐test	Post‐intervention test	Follow‐up test	*χ* ^2^	*p*
Paraffin	2.3 ± 2.0	3.6 ± 1.7	4.7 ± 1.7	3.1 ± 1.3	52.05	<0.001*
JB4	1.8 ± 1.6	3.8 ± 1.3	6.0 ± 2.1	5.7 ± 1.3	87.47	<0.001**
All students	2.1 ± 1.8	3.7 ± 1.5	5.4 ± 2.0	4.3 ± 1.8	102.90	<0.001***

*Note*: Within‐group time‐dependent progression was tested using the Friedman analysis. Post‐hoc differences were evaluated with the Wilcoxon signed‐rank test.

* There was no significant difference between the pre‐test and the follow‐up test (*p* = 0.509), whereas the comparisons between the other groups showed significant differences (*p* < 0.001).

** There was a significant difference among all groups (*p* < 0.001).

*** All comparisons were significant (*p* < 0.001), except for the comparison between the immediate post‐test and the follow‐up test (*p* = 0.615).

Specifically, the Pre‐Test, Immediate Post‐Test, Post‐Intervention Test, and Follow‐Up Test each consisted of the same 10 multiple‐choice questions. The calculated difficulty indices for these assessments were 0.41, 0.32, 0.45, and 0.49, respectively. According to established benchmarks (≈0.3–0.5), these values indicate that the tests were of moderate difficulty. A detailed analysis of the difficulty indices across the different assessments is provided in Table 3.

**TABLE 3:** Difficulty index results of the exams.

Exams	Median score	Difficulty index
Pre‐test	2.0	0.41
Immediate post‐test	4.0	0.32
Post‐intervention test	5.0	0.45
Follow‐up test	4.0	0.49

Participants were also grouped by gender (female: *n* = 169, male: *n* = 128), and their exam results were compared. Since the assumption of normal distribution was not met, the nonparametric Mann–Whitney *U* test was applied, and effect sizes were calculated using the Rank–Biserial Correlation (*r*_s_). In the Pre‐Test (*r*_s_ = −0.07), Immediate Post‐Test (*r*_s_ = −0.09), Post‐Intervention Test (*r*_s_ = −0.12), and Follow‐Up Test (*r*_s_ = 0.02), only small effect sizes were observed between genders. However, in the practical exam, a statistically significant difference was found between male and female students (*p* = 0.001), with an effect size of *r*_s_ = −0.22, indicating a small‐to‐moderate effect. The median practical exam score of female students was 5.7 (IQR = 3.5; min = 0.25, max = 9.0), whereas that of male students was 4.5 (IQR = 0.25; min = 0.25, max = 8.7).

Furthermore, the effects of gender (female/male) and group (Paraffin/JB4) variables on exam outcomes were examined. While both gender and group demonstrated varying levels of influence across different assessments, no significant gender × group interaction was observed in any of the tests (*p* > 0.05). The results are presented in Table 4.

**TABLE 4:** The effects of gender (female/male) and group (Paraffin/JB4) variables on exam outcomes.

Assessment	Gender *p*	Group *p*	Gender × Group *p*
Pre‐test	0.383	–	–
Immediate post‐test	0.177	–	–
Post‐intervention test	0.119	<0.001	0.428
Follow‐up test	0.935	<0.001	0.061
Drawing‐based performance assessment	0.002	0.105	0.572

Finally, open‐ended questions were analyzed through interviews with 22 students. When asked about the advantages of the JB4 block technique, students most frequently emphasized aspects related to visual quality, using terms such as “clarity,” “structural integrity of cells,” “visibility of details,” and “thin sections.” Reported disadvantages included “difficulty of the preparation process for instructors,” “complexity due to excessive detail,” “challenges in interpreting some structures because they appear differently compared to paraffin,” and “higher cost.” In terms of preference, the majority of participants indicated that they favored the JB4 block over paraffin, as lessons using JB4 provided greater detail and clarity.

## DISCUSSION

Histology education has a history spanning more than 200 years. Advances in tissue preparation techniques have played a pivotal role in its evolution, enabling detailed analysis of tissues and facilitating a deeper understanding of structures.^15^ Nevertheless, histology remains a challenging subject for students due to the need to interpret intricate cellular‐level structures, which imposes a considerable cognitive load. The quality of instructional materials is therefore critical, as it can directly affect learning outcomes. Recently, the integration of digital technologies into medical education has been emphasized in the literature as a means of enhancing the quality of educational resources. A multidimensional evaluation of how digital tools influence learning is necessary. To the best of our knowledge, this is the first study to comparatively examine the effect of histologic sections prepared with the JB4 plastic resin technique on learning in a digital environment. However, an additional methodological factor that may have influenced student perspectives is the difference in staining protocols applied to paraffin and JB‐4 sections. While paraffin sections were stained with H&E, JB‐4 sections were stained using Fuchsin–Toluidine Blue, which provides enhanced nuclear and cytoplasmic contrast. As demonstrated in our recent comparative study,^14^ JB‐4 embedding is also compatible with PAS and reticulin staining, enabling improved visualization of glycoconjugates, basement membranes, and reticular fiber networks. These staining‐related enhancements may contribute to improved structural recognition in virtual microscopy‐based learning environments.

Beyond comparisons with routine paraffin embedding, illustrated in Figure 2, it is also important to contextualize JB‐4 embedding relative to other plastic‐based histological preparation techniques reported in the literature. Various plastic embedding systems, including epoxy resins and alternative methacrylate‐based media, have been used to obtain high‐resolution histological sections, particularly for ultrastructural or specialized applications. However, many of these methods require complex processing steps, have limited staining compatibility, or are less suited for routine light microscopy and educational use.

In contrast, JB‐4 (glycol methacrylate‐based) embedding enables the preparation of semi‐thin sections with enhanced cellular resolution while remaining compatible with conventional light microscopy and a wide range of histochemical stains. As demonstrated in our previous comparative work,^14^ JB‐4 sections provide improved nuclear and cytoplasmic detail compared with paraffin sections and offer practical advantages over other plastic embedding techniques in terms of staining flexibility and suitability for digital slide scanning. These features make JB‐4 particularly well suited for virtual microscopy‐based histology education, where high image clarity and structural recognizability are critical.

In our study, the Post‐Intervention Test scores of students in the JB4 group were significantly higher than those in the paraffin group. This finding is consistent with previous research demonstrating that improved image quality enhances student performance in digital environments. For instance, Başer and Büyük^6^ reported that virtual microscopy was associated with superior performance outcomes compared to light microscopy among medical students. Similarly, a study from the University of Michigan revealed that medical and dental students preferred digital methods over traditional ones in histology education, with virtual applications improving learning outcomes.^2^ A meta‐analysis further confirmed that medical students favor virtual microscopy, which has a positive effect on exam performance.^5^ Collectively, these findings support the conclusion that as the visual quality of histologic sections improves, so too does learning performance.

No statistically significant differences were observed between the JB4 and paraffin groups in drawing‐based performance. However, gender‐based analyses revealed that female students outperformed male students. This aligns with literature highlighting gender differences in spatial task performance: males typically perform better in tasks requiring mental spatial rotation, whereas females tend to excel in fine motor skills.^16^ Such differences may have influenced performance in the drawing‐based exam, where fine motor ability is particularly relevant. Although no significant gender differences were observed in knowledge retention, the superior fine motor skills of female students may explain their advantage in the practical assessment. In histology, spatial rotation skills are essential for interpreting microscope images, modeling tissue structures from different angles, and understanding cross‐sectional planes. While gender was not a primary focus of our study, these findings highlight that individual learning and self‐expression vary across sociodemographic characteristics.^17^ Consequently, providing diverse learning and assessment tools may help accommodate different learning needs.

Quantitative analyses in our study indicate that histological sections prepared using the JB4™ technique significantly improve student learning outcomes, particularly in terms of visual clarity, cellular detail, and knowledge retention. In qualitative evaluations, students emphasized advantages such as clarity, structural integrity of cells, and the visibility of fine details. While paraffin embedding and sectioning remain widely used and generally adequate, preparing semi‐thin sections with paraffin is technically challenging, and artifacts can limit cellular resolution. In contrast, plastic resin embedding techniques such as JB4™ allow straightforward preparation of ultrathin (0.5–1 μM) or semi‐thin (2–3 μM) sections.^18^ Thin sections have long been recognized as providing superior cytological detail compared to thicker ones, thereby enabling students to better understand tissue differences by visualizing microscopic structures with greater fidelity and less distortion.

Despite these advantages, JB4 preparation also poses challenges. It requires substantial technical expertise and time investment by instructors, and the high cost of the material, along with limited availability across laboratories, constrains its widespread use. A practical solution may involve digitizing JB4 preparations into high‐resolution virtual slides, reducing costs while ensuring continued access to high‐quality visual content. Virtual microscopy has already begun transforming histology education, with many medical schools integrating or planning to adopt this technology.^6^, ^7^ Although some institutions have transitioned fully to computer‐assisted learning, students have expressed concerns that exclusive reliance on digital systems may limit the development of essential microscopy skills for professional practice.^6^, ^19^, ^20^ Therefore, integrating digital resources with traditional paraffin‐based microscopy offers a balanced approach, preserving practical skills while enhancing visual learning through digital innovations. This approach aligns with mixed teaching models recommended in the literature.^6^, ^7^

## CONCLUSION

Our findings suggest that the use of JB4 plastic resin sections in histology education may provide advantages over traditional paraffin sections, particularly in terms of post‐intervention performance and knowledge retention. While no significant differences were observed in drawing‐based performance, qualitative feedback indicated that students perceived JB4 sections as clearer and more detailed, although preparation difficulties and higher costs were noted as limitations. These results highlight that incorporating JB4‐derived materials, especially in digital formats, could complement conventional paraffin‐based methods and contribute to enhancing the quality of histology education in a feasible and balanced way.

## LIMITATION

Low follow‐up participation: The relatively low participation rate in the Follow‐Up Test (25.2%) may limit the generalizability of the findings regarding knowledge retention and introduce the risk of attrition bias.

Focus group only with JB4 group: Qualitative data were obtained only from the JB4 group, since these students had experienced both paraffin and JB4 preparations. However, the lack of feedback from the paraffin group restricts direct comparison of student perspectives between groups.

Single center and population: The study was conducted in a single medical school with second‐year medical students. Therefore, the external validity of the findings is limited, and results may not be generalizable to other institutions or student groups.

Scope of histological content: The study was limited to epithelial and selected tissue types. Findings may not fully reflect the outcomes of histology education across all tissue structures.

Fixed test items: The same 10 multiple‐choice questions were used across all assessment points, which may have introduced test–retest or recall effects, particularly in the immediate post‐test, potentially inflating short‐term performance gains. This approach was chosen to ensure direct comparability of scores over time and to evaluate learning gains and retention using identical content. However, repeated exposure to the same items may have influenced students' performance independently of true learning. To partially address this limitation, a drawing‐based performance assessment was also included, which required students to recall and label histological structures rather than rely solely on recognition.

Different staining protocols: Different staining protocols were used for paraffin (H&E) and JB‐4 (Fuchsin–Toluidine Blue) sections. Although JB‐4 embedding offers superior section quality and compatibility with additional histochemical stains such as PAS and reticulin, the independent effects of staining‐related contrast cannot be fully separated from those of the embedding medium. Future studies applying identical staining protocols across embedding methods may help to clarify these effects.

Uncontrolled exposure to review materials: Participants' exposure to self‐directed review or study materials between the post‐intervention test and the follow‐up assessment was not formally assessed. Although no additional teaching sessions or structured review activities were provided during this interval, students' individual study behaviors were not monitored. Therefore, performance observed in the follow‐up test may have been influenced not only by the instructional intervention but also by independent review, which cannot be fully ruled out.

Implementation constraints: While JB4 provides superior image quality, its higher preparation cost and technical workload limit the feasibility of large‐scale application. Thus, sustainability and accessibility remain challenges for broader adoption.

## AUTHOR CONTRIBUTIONS

**Zeynep Deniz Şahin İnan:** Conceptualization; investigation; writing – original draft; methodology; validation; writing – review and editing; formal analysis; project administration; supervision; resources; visualization. **Ezgi Ağadayı:** Conceptualization; investigation; writing – original draft; methodology; visualization; writing – review and editing; software; formal analysis; supervision.

## FUNDING INFORMATION

This research did not receive any specific grant from funding agencies in the public, commercial, or not‐for‐profit sectors.

## CONFLICT OF INTEREST STATEMENT

The authors declare that they have no competing interests.

## ETHICS STATEMENT

The study was approved by Sivas Cumhuriyet University Health Sciences Research Ethics Committee (Protocol No: 2024/09‐42; Date: 19.09.2024). Written informed consent was obtained from all participants.

## Supporting information


Data S1.

## Data Availability

The datasets generated and analyzed during the current study are available from the corresponding author upon reasonable request.
