# Supplementary material for: Comparison of JB4 plastic resin and standard paraffin methods on student performance and student perspectives in digital histology education: A randomized controlled study
Source: Anat Sci Educ. 2026 Mar 8;19(5):723–31. doi: 10.1002/ase.70213 (PMC13184576; doi:10.1002/ase.70213)
Supplement: Supplementary file 1 — Data S1. [file ASE-19-723-s001.pdf]

The following questions were used in the Pre-Test, Immediate Post-Test, Post-Intervention Test, and Follow-Up Test. The correct answer to each question is indicated by (**).

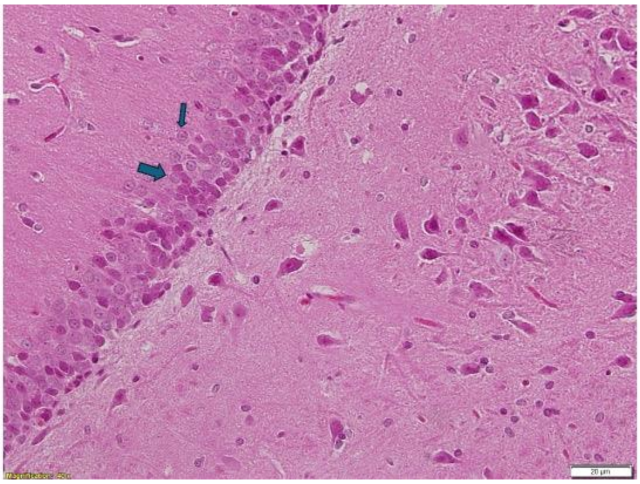

Identify the area indicated by the blue arrow in the histological preparation above

A) Astrocyte
B) Oligodendrocyte
C) Purkinje cell **
D) Granule cell
E) Microglia

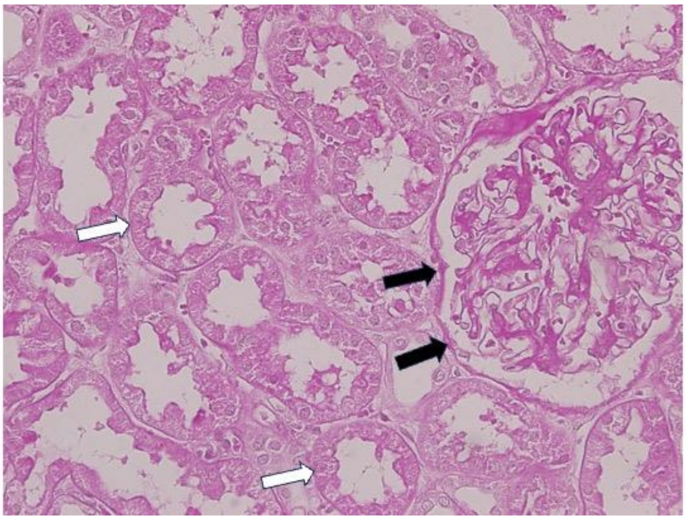

Identify the areas indicated by the black and white arrows in the histological preparation above

A) White: simple columnar epithelium – Black: simple cuboidal epithelium
B) White: simple squamous epithelium – Black: simple cuboidal epithelium
C) White: simple cuboidal epithelium – Black: simple squamous epithelium **
D) White: stratified squamous epithelium – Black: simple columnar epithelium
E) White: stratified cuboidal epithelium – Black: stratified squamous epithelium

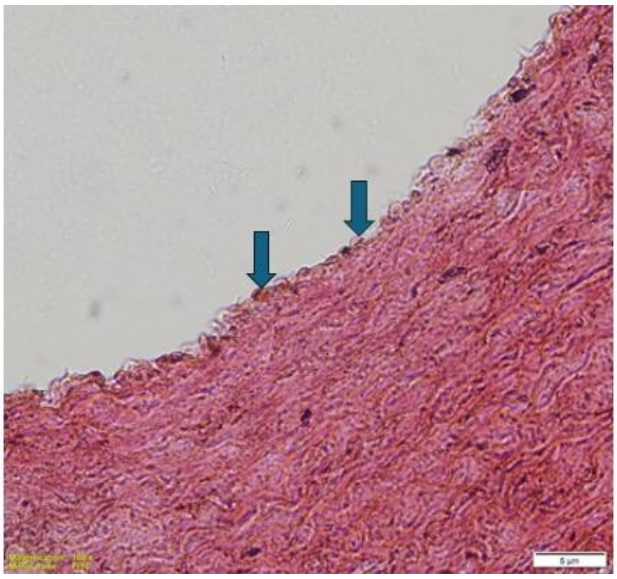

The cells indicated by the arrows most likely represent which of the following cell types?

A) Simple squamous epithelial cells (endothelium) **
B) Simple cuboidal epithelial cells
C) Fibroblasts
D) Smooth muscle cells
E) Mesenchymal stellate cells

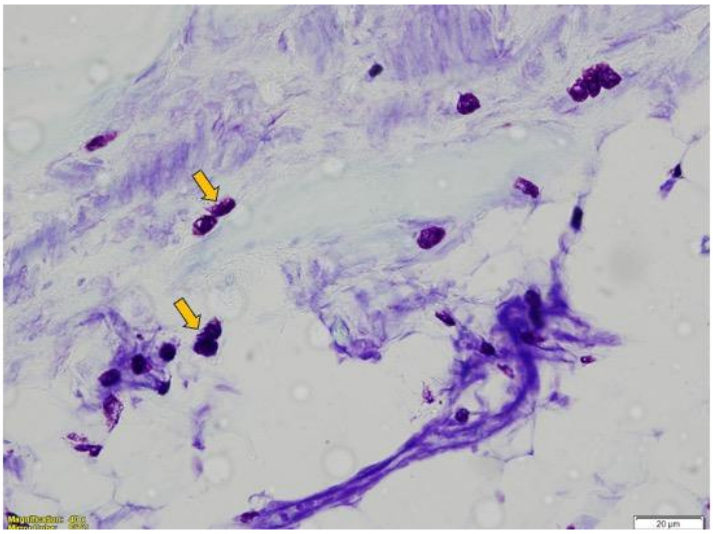

In the histological section shown, the cells indicated by the arrows are most likely which of the following?
A) Fibroblasts
B) Plasma cells
C) Mast cells **
D) Macrophages
E) Adipocytes

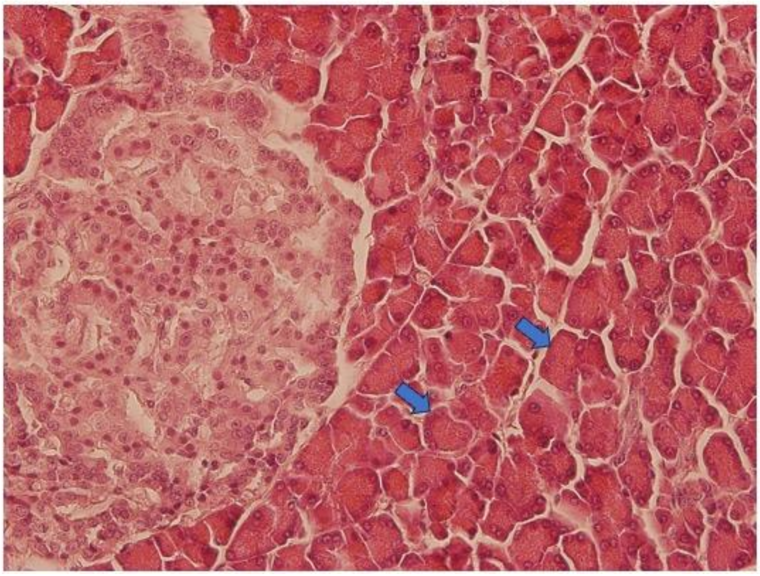

In the histological section shown, the cells indicated by the arrows are most likely which of the following?
A) Endocrine cells of the islets of Langerhans
B) Ductal epithelial cells
C) Pancreatic acinar cells **
D) Hepatocytes
E) Smooth muscle cells

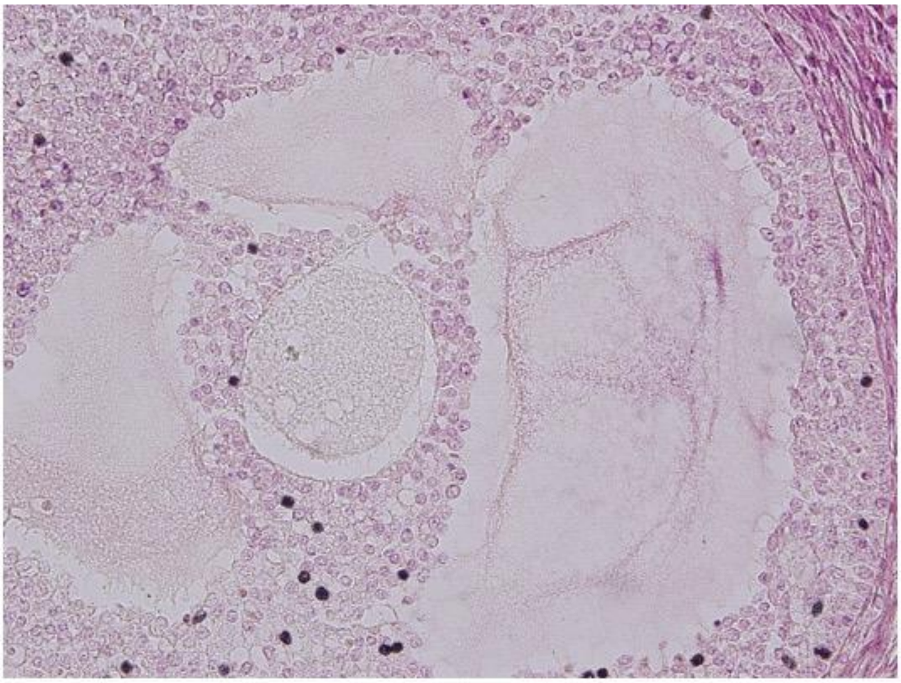

In the histological section shown, which structure is most likely represented?
A) Primary ovarian follicle
B) Secondary (antral) ovarian follicle **
C) Corpus luteum
D) Corpus albicans
E) Seminiferous tubule

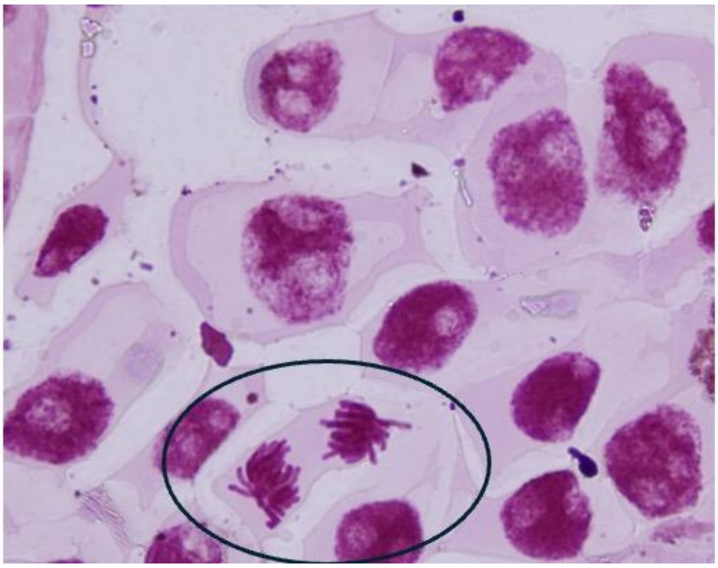

Identify the stage of mitosis indicated by the circled area

A) Prophase
B) Anaphase **
C) Metaphase
D) Telophase
E) Interphase

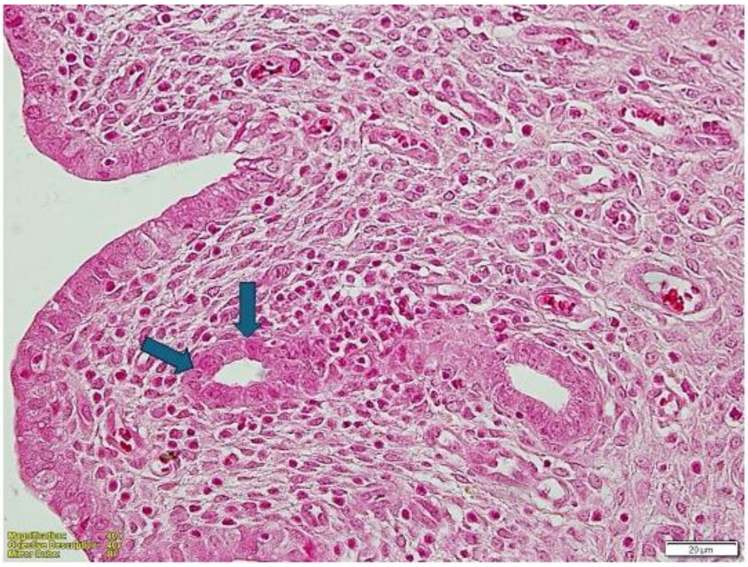

Identify the area indicated by the blue arrow in the histological preparation above

A) Acinus
B) Follicle
C) Blood vessel
D) Endometrial gland **
E) Nerve ending

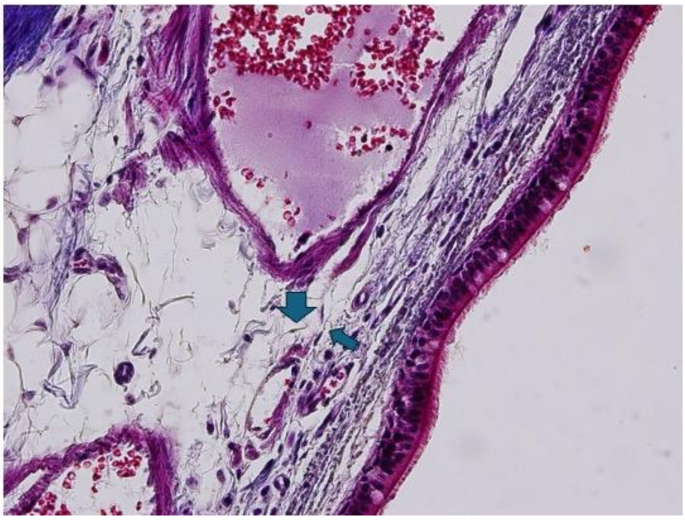

Identify the area indicated by the blue arrow in the histological preparation above

A) Endothelium **

B) Mesenchyme

C) Erythrocyte

D) Mast cell

E) Fibroblast

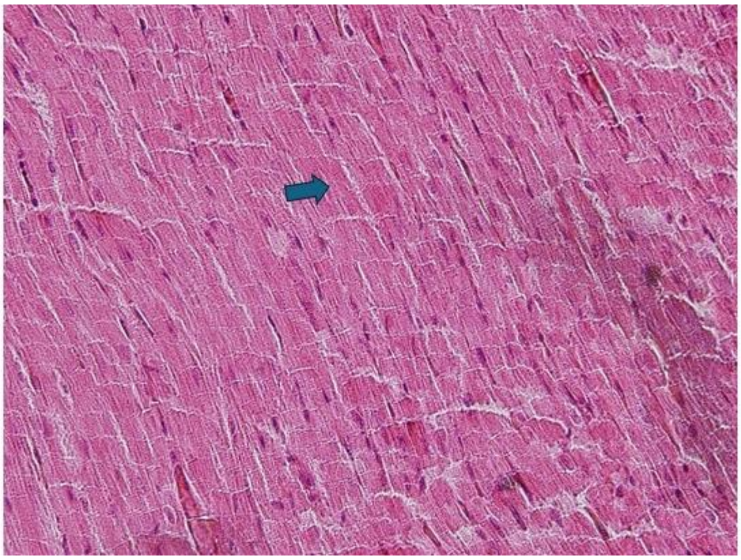

Identify the area indicated by the blue arrow in the histological preparation above

A) Cardiac muscle cell
B) Smooth muscle cell
C) Skeletal muscle cell **
D) Fibroblast
E) Neuron
